# Gain-Phase Errors Calibration for a Linear Array Based on Blind Signal Separation

**DOI:** 10.3390/s20154233

**Published:** 2020-07-29

**Authors:** Zheng Dai, Weimin Su, Hong Gu

**Affiliations:** School of Electronic Engineering and Optoelectronic Technology, Nanjing University of Science and Technology, Nanjing 210000, China; daizheng@njust.edu.cn (Z.D.); guhong666@njust.edu.cn (H.G.)

**Keywords:** linear array, gain-phase error, array error calibration, DOA estimation

## Abstract

In this paper, a non-iterative blind calibration algorithm for gain-phase errors is proposed. A mixing matrix is first obtained from the received observation data through blind signal separation. The mixing matrix is the product of the gain-phase error matrix and the ideal array manifold matrix. Then, a spatial spectrum is constructed by using the estimated mixed matrix. The direction corresponding to the maximum point of the spectral function is proved to be the azimuth of a certain source. Therefore, after the direction-of-arrival (DOA) is obtained by a one-dimensional spectrum search, the active calibration method can be used to estimate the gain-phase errors. The proposed algorithm is not limited to the calibration for uniform linear array (ULA), but also applicable to a non-uniform linear array. Moreover, the estimation performance of the algorithm will not be affected by the magnitude of the gain errors. Some simulations are given to verify the effectiveness and performance of the algorithm.

## 1. Introduction

Direction-of-arrival (DOA) estimation is an important research field in array signal processing, which is widely used in radar, sonar, communication, biomedical and other fields [1,2,3,4,5]. However, most of the high-resolution estimation algorithms [6,7,8,9,10] are based on the premise that the position of the array element, the amplitude and phase response of the channel and the mutual coupling effect between the array elements are accurately known. However, in the practical engineering application, due to the inevitable errors, the actual array manifold often appears a certain degree of deviation, at this time the performance of the commonly used high-resolution estimation algorithm will be seriously deteriorated [11,12]. In order to realize the practicality of high-resolution DOA estimation algorithms, it is necessary to calibrate the sensor array errors. The amplitude and phase response between channels, called gain-phase error, is considered in this paper.

One method is to calibrate the sensor array error by setting one or more calibration sources with accurately known positions in the near-field or far-field space [13,14,15,16]. The method in Reference [13] presents a simple expression of the log-likelihood function and gives some mathematical properties of the objective function, and then sensor gain and phase errors are estimated when the true field covariance at the sensor location is known exactly. In Reference [14], three well-known algorithms: conditional maximum likelihood (CML), unconditional maximum likelihood (UML), and weighted noise subspace fitting (WNSF) are used to estimate gain-phase errors. In terms of estimation performance, the WNSF algorithm and UML algorithm are more effective than the CML algorithm. In Reference [15], the gain-phase errors and geometry of the array sensor are estimated by using a set of simultaneous equations formed by music spatial spectrum characteristics, and all the DOAs of the signal sources are required to be known. Since the method has no iterative operation, the convergent problem does not need to be considered. In Reference [16], the EACDM algorithm and EACIM algorithm are proposed for the conventional data model and improved data model, respectively. The two algorithms perform independently of the phase error and need a small amount of computation. The active calibration algorithm is widely used in practical engineering because it does not need DOA estimation and the calculation is very small. However, this kind of method has high accuracy requirements for the location and azimuth of auxiliary signal sources [17].

Different from the active calibration methods, the blind calibration algorithm can estimate the DOA and sensor array errors online without setting auxiliary signal sources. The methods in References [18,19,20,21] model the DOA estimation in the presence of gain-phase errors as a quadratic optimization problem, and the array error is compensated by solving the optimization problem. The precondition of the methods is that some transceiver array elements have been strictly calibrated. The methods in References [22,23] is applicable to transceiver arrays with more than two calibrated array elements, and the DOA estimation does not require pre-calculation of gain-phase errors. References [24,25] define a cost function based on the principle of subspace orthogonality, and iterates between the direction and the array error to obtain the minimum point as an estimate of the array error and DOA. The performance of the algorithms depends on the initial value of the iteration. When the initial value is far from the real value, the algorithm cannot get an effective calibration effect. Reference [26] analyzes the applicable conditions of the gain-phase errors blind calibration algorithm in Reference [24], and points out that the algorithm is not suitable for the case where the number of elements is less than or equal to 4. In addition, there are special geometric arrangements and direction combinations, which make the phase error estimation ambiguous. Similar to the DOA estimation problem, subspace fitting algorithms can also be applied to array error calibration problems, References [27,28] use the maximum likelihood, weighted signal subspace, and weighted noise subspace fitting principles to construct a multidimensional nonlinear cost function. This algorithm is universal to many forms of array error, and has high estimation performance, but it is similar to the subspace fitting class DOA estimation algorithm, which has high computational complexity. In order to solve the high-dimensional nonlinear optimization problem, some papers [29,30,31] in recent years have applied modern optimization algorithms to array blind calibration, to a certain extent, solving the problems of local convergence and high computational complexity of the algorithm. In general, the cost function minimization algorithm has strong applicability and high calibration performance for different types of errors. In theory, many algorithms can obtain optimal estimation performance. The disadvantage of these algorithms is that the calculation complexity is high, and it is difficult to select the initial value of the iterative algorithm. The research of independent component analysis (ICA) method [32] expands the idea of array error parameter estimation. In Reference [33], the received signals from different sources are separated by the ICA method, and then the DOAs of signal sources and mutual coupling coefficients of array elements are estimated jointly. In Reference [34], the ICA method is used to estimate the gain-phase errors and the array element position errors. This method can estimate the error parameters when the number of sources is larger than the number of array elements, but it can only be used for ULAs at present.

In recent years, there is a kind of popular algorithm we call a “decoupling” algorithm. As the name implies, it is to eliminate the coupling between unknown parameters, so that the solution of a group of unknown parameters is not affected by other unknown parameters. The main idea is to process the array observation data to eliminate the array error term, so it can be directly used to estimate the direction of the signal source, and then the array error can be calibrated by using the active algorithm. Based on this idea, a blind gain-phase errors calibration algorithm based on eigenvalue decomposition is proposed in References [35,36], the square of the array output signal amplitude is extracted to calculate the covariance matrix. The covariance matrix does not contain the phase error, so the subspace algorithm can be directly used to estimate the direction of the signal source. In References [37,38], the relationship between the gain-phase errors and the array manifold vector is established to eliminate the errors in MUSIC spatial spectrum, Then the DOA of two calibration sources is estimated by two-dimensional spatial spectrum search. The algorithms in References [35,36,37,38] do not need iteration, and the estimation performance is independent of the phase error. It is also suitable for large phase error. The disadvantage is that there are many requirements for the array, and two sources that are relatively far away in the spatial direction are required.

In this paper, the joint approximation and diagonalization of eigenmatrices (JADE) algorithm [39] is implemented to obtain a mixing matrix, which is the array manifold matrix adjusted by gain-phase errors. In order to eliminate the DOA information in the mixing matrix, we construct a spatial spectrum and find that when the direction variable is equal to the DOA of the signal, the spatial spectrum should be a specific value related to the phase errors. We prove that this specific value is the maximum value of the spectral function under the assumptions that the expectation of phase error is zero and the number of elements is large enough, so the gain-phase errors can be easily estimated after the DOA is obtained by one-dimensional search over the spectral function. The proposed algorithm can calibrate the gain-phase errors of the non-uniform linear array, and the estimation accuracy is independent of the magnitude of the phase errors.

## 2. Data Model

**Figure 1:**
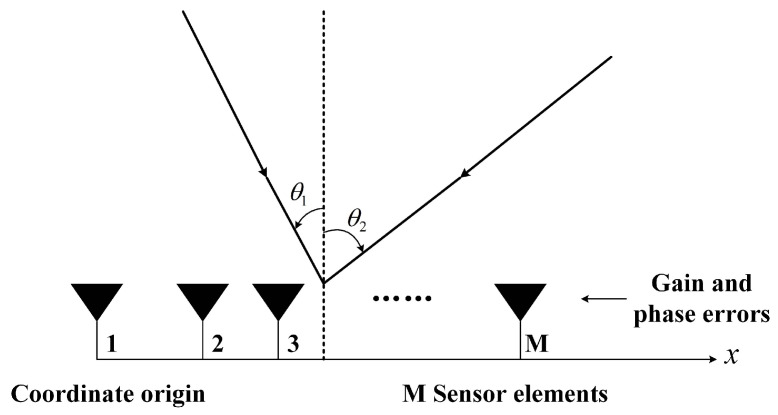
A linear array and multiple sources with unknown direction-of-arrival (DOAs).

As shown in Figure 1, consider a linear array consisting of M omnidirectional array elements, where the first array element is used as a reference array element. The data model is simplified in this case, but can be applied to sensors without omni-direcitonal patterns. The distance between the *i*-th array element and the first array element is $d_{i}$. Assume there are *D* narrowband signals emitted by the far-field sources impinging on this linear array. Then the vector of observation data can be expressed as [10]

(1) $$x\left(t\right)=\Gamma \mathrm{As}\left(t\right)+n\left(t\right)\;,$$

where n(t) is a vector of zero-mean white additive noise. The signal vector $s\left(t\right)={\left[s_{1}\left(t\right),s_{2}\left(t\right),\ldots s_{D}\left(t\right)\right]}^{T}$ is composed of *D* incident signals in different directions. Here, ${\left[\cdot \right]}^{T}$ indicates the transpose operation. Define an M×M diagonal matrix as the gain-phase error matrix, which is given by

(2) $$\Gamma ≜diag\left(\left[1,\rho_{2}e^{j\varphi_{2}},\ldots ,\rho_{M}e^{j\varphi_{M}}\right]\right)\;,$$

where $\rho_{i}$ and $\varphi_{i}$ are the gain and phase errors of the *i*-th sensor element, respectively. As the first sensor is the reference sensor, we have $\rho_{1}=1$ and $\varphi_{1}=0$. Being the same as References [35,36,37], $\rho_{i}$ and $\varphi_{i}\;\left(i=2,3,\ldots ,M\right)$ are independent distributed random variables, and the mathematical expectations can be expressed as

(3) $$\begin{array}{c} E\left(\rho \right)=1,\\ E\left(\varphi \right)=0. \end{array}$$

Furthermore, $A=[a\left(\theta_{1}\right),a\left(\theta_{2}\right),\ldots ,a\left(\theta_{D}\right)]$ denotes the M×D array manifold matrix. $a(\theta_{i})$ is the ideal array manifold vector at direction $\theta_{i}$, which is formulated by

(4) $$a\left(\theta_{i}\right)={\left[1,e^{-j\frac{2\pi }{\lambda }d_{2}\sin\theta_{i}},\ldots ,e^{-j\frac{2\pi }{\lambda }d_{M}\sin\theta_{i}}\right]}^{T}\;,$$

where λ is the center wavelength. Based on Equation (1), we define the M×D array manifold matrix adjusted by gain-phase errors as a mixing matrix, which is given by

(5) $$B≜\left[b\left(\theta_{1}\right),b\left(\theta_{2}\right),\ldots ,b\left(\theta_{D}\right)\right]=\Gamma A.$$

Without loss of generality, we suppose that the directions of all the incident signals are different. Moreover, the signals which are non-Gaussian in distribution, are independent of each other and independent of noise. We consider the gain-phase errors is constant over time, and the fluctuations with frequency can be ignored in the measurement period, which means the sensor gain-phase errors will not drift over our measurement interval.

## 3. Proposed Algorithm

### 3.1. Mixing Matrix Estimation

As a traditional blind signal separation algorithm, the JADE algorithm can separate the incident signal and the array manifold matrix with high estimation accuracy and fast convergence. In this letter, we separate the vector s(t) and the matrix **B** by making use of the JADE algorithm. The detailed algorithm implementation is described in Reference [39]. The estimated mixing matrix is expressed as

(6) $${\hat{B}}_{JADE}=\left[b_{J}\left(\theta_{1}\right),b_{J}\left(\theta_{2}\right),\ldots b_{J}\left(\theta_{D}\right).\right]\;$$

Since the first array element located at the coordinate origin is the reference array element. The M×D matrix ${\hat{B}}_{JADE}$ can be normalized as

(7) $$\begin{array}{cc} \hat{B} & =\left[\hat{b}\left(\theta_{1}\right),\hat{b}\left(\theta_{2}\right),\ldots ,\hat{b}\left(\theta_{D}\right)\right]\\ \; & =\left[\frac{b_{J}\left(\theta_{1}\right)}{{\left[b_{J}\left(\theta_{1}\right)\right]}_{1}},\frac{b_{J}\left(\theta_{2}\right)}{{\left[b_{J}\left(\theta_{2}\right)\right]}_{1}},\ldots ,\frac{b_{J}\left(\theta_{D}\right)}{{\left[b_{J}\left(\theta_{D}\right)\right]}_{1}}\right]\;, \end{array}$$

where ${\left[\cdot \right]}_{i}$ denote the *i*-th element of the vector. It is worth noting that the JADE algorithm permutes the order of the vectors in **B**, however, we only randomly use one of these vectors for DOA estimation. In addition, the incident signals are separated as $\hat{s}\left(t\right)={\hat{V}}^{H}\hat{Y}x\left(t\right)$, where ${\left(\cdot \right)}^{H}$ denotes the conjugate transpose. Here, $\hat{V}$ is a unitary matrix and $\hat{Y}$ is a whitening matrix. They are estimated by using the JADE algorithm under the assumption that the number of source signals is known, and is small relative to the number of sensors.

### 3.2. Doa Estimation

It can be found from Equation (5) that the estimated mixing matrix contains error information and DOA information. In order to eliminate the DOA information, we first introduce a vector,

(8) $$a\left(\gamma \right)={\left[1,e^{-j\frac{2\pi }{\lambda }d_{2}\sin\gamma },\ldots ,e^{-j\frac{2\pi }{\lambda }d_{M}\sin\gamma }\right]}^{T}\;.$$

Here, γ is an unknown direction variable, −π/2≤γ≤π/2. In order to eliminate DOA information as much as possible, we construct a function which is defined as

(9) $$f\left(\gamma \right)≜\sum_{i=2}^{M}{\left\{\arg\left(\frac{{\left[\hat{b}\left(\theta_{1}\right)\right]}_{i}}{{\left[a\left(\gamma \right)\right]}_{i}}\right)\right\}}^{2}\;,$$

where arg(·) denotes the argument of complex numbers. It is obvious that when γ is equal to the direction $\theta_{1}$, we have $f\left(\gamma \right)=\varphi_{2}^{2}+\ldots +\varphi_{M}^{2}$. At this time, only the phase error information is included in the function value. In order to estimate the direction $\theta_{1}$, we need to investigate whether this specific value has particularity in the spectral function. Insert Equation (8) into Equation (9), the function can be rewritten as

(10) $$f\left(\gamma \right)=\sum_{i=2}^{M}{\left[\varphi_{i}+2\pi d_{i}\left(\sin\gamma -\sin\theta_{1}\right)/\lambda \right]}^{2}\;.$$

In order to facilitate the mathematical derivation, we suppose w=sinγ, the function can be simplified as

(11) $$f\left(w\right)=\sum_{i=2}^{M}{\left[\varphi_{i}+2\pi d_{i}\left(w-\sin\theta_{1}\right)/\lambda \right]}^{2}\;.$$

The minimum point of the function f(w) is obtained by calculating the root of the equation $f^{\prime }\left(w\right)=0$, which is given by

(12) $$w_{0}=-\frac{\lambda \sum_{i=2}^{M}d_{i}\varphi_{i}}{2\pi \sum_{i=2}^{M}d_{i}^{2}}+\sin\theta_{1}\;.$$

The expectation and variance of $w_{0}$ can be expressed as

(13) $$E\left(\omega_{0}\right)=E\left(-\frac{\lambda \sum_{i=2}^{M}d_{i}\varphi_{i}}{2\pi \sum_{i=2}^{M}d_{i}^{2}}+\sin\theta_{1}\right)=\sin\theta_{1}$$

(14) $$\begin{array}{cc} D\left(w_{0}\right) & =D\left(-\frac{\lambda \sum_{i=2}^{M}d_{i}\varphi_{i}}{2\pi \sum_{i=2}^{M}d_{i}^{2}}+\sin\theta_{1}\right)\\ \; & =\frac{\lambda^{2}}{\left(M-1\right){\left(2\pi \right)}^{2}\sum_{i=2}^{M}d_{i}^{2}}\sigma_{\varphi }^{2}\;, \end{array}$$

where $\sigma_{\varphi }^{2}$ is the variance of phase error. From Equation (14), the variance of $w_{0}$ decreases as the number of sensor elements *M* increases and the variance of phase errors $\sigma_{\varphi }$ decreases. From Equation (13), the mathematical expectation of $w_{0}$ is equal to $\sin\theta_{1}$. This means that when *M* is large enough, DOA can be obtained by finding the minimum value of $f\left(\gamma \right)$.

(15) $$\begin{array}{c} f\left(\gamma \right)≜\sum_{i=2}^{M}{\left\{\arg\left(\frac{{\left[\hat{b}\left(\theta_{1}\right)\right]}_{i}}{{\left[a\left(\gamma \right)\right]}_{i}}\right)\right\}}^{2}\;,\\ {\hat{\theta }}_{1}=\arg\min_{\gamma }f\left(\gamma \right),\;\gamma \in \left[-\frac{\pi }{2},\frac{\pi }{2}\right]\;. \end{array}$$

Define

(16) $$\Delta \hat{b}\left(\theta_{i}\right)≜{\left[\frac{{\left[\hat{b}\left(\theta_{i}\right)\right]}_{2}}{{\left[\hat{b}\left(\theta_{1}\right)\right]}_{2}},\ldots ,\frac{{\left[\hat{b}\left(\theta_{i}\right)\right]}_{M}}{{\left[\hat{b}\left(\theta_{1}\right)\right]}_{M}}\right]}^{T}\;.$$

The *m*-th element of the vector on both sides of Equation (16) can form an equation, which is given by $2\pi d_{m}\left(\sin\theta_{i}-\sin\theta_{1}\right)/\lambda =\arg\left({\left[\Delta \hat{b}\left(\theta_{i}\right)\right]}_{m}\right)$. In matrix form

(17) $${\left[d_{2},\ldots d_{M}\right]}^{T}\cdot \left(\sin\theta_{i}-\sin\theta_{1}\right)=\frac{\lambda \arg\left(\Delta \hat{b}\left(\theta_{i}\right)\right)}{2\pi }\;.$$

Equation (17) indicates that M−1 equations are obtained for estimating $\sin\theta_{i}$, and we take the average of them as the estimated value,

(18) $$\sin\theta_{i}=\frac{1}{M-1}\left\{\left[\frac{\lambda }{2\pi d_{2}}\arg\left({\left[\Delta \hat{b}\left(\theta_{i}\right)\right]}_{2}\right)+\sin\theta_{1}\right]+\cdots +\left[\frac{\lambda }{2\pi d_{M}}\arg\left({\left[\Delta \hat{b}\left(\theta_{i}\right)\right]}_{M}\right)+\sin\theta_{1}\right]\right\}\overset{\Delta }{\;=}k_{i}\;.$$

Thus, the DOA of the *i*-th signal can be estimated numerically from ${\hat{\theta }}_{i}=\arcsin k_{i},\;i=2,3,\ldots D$.

### 3.3. Gain-Phase Errors Estimation

According to the estimated mixing matrix $\hat{B}$ and the estimated DOA ${\hat{\theta }}_{i}$, the gain-phase errors matrix is obtained as

(19) $$\Gamma =\frac{1}{D}\sum_{i=1}^{D}diag\left[\hat{b}\left(\theta_{i}\right)⊙a^{*}\left({\hat{\theta }}_{i}\right),\right]$$

where the superscripts ⊙ and ${\left(\cdot \right)}^{*}$ represent the Hadamard product and conjugate operation, respectively. The gain errors and phase errors can be calculated as

(20) $$\begin{array}{c} \begin{array}{c} \rho_{m}=\left|\Gamma_{mm}\right|\;\\ \varphi_{m}=\arg\left(\Gamma_{mm}\right), \end{array} \end{array}$$

where $\left|\cdot \right|$ indicates the modulus of a complex number.

## 4. Discussion

In this paper, we compare the computational complexity of the proposed method, the method in Reference [40] and the EACDM method. Suppose that the number of array elements is *M*, the number of sources is *D*, the number of snapshots is *L*, and the number of search points is *N*. Then, the computational complexity of the proposed method is $O\left(M^{3}+MD^{2}+D^{4}L+MN\right)$, the computational complexity of the method in Reference [40] can be expressed as $O\left(M^{3}+M^{2}L+MN\right)$, and the computational complexity of the EACDM method can be described as $O\left(3ML\right)$. It can be seen that the EACDM method has the lowest computational complexity. The computational complexity of the proposed algorithm is more affected by the number of sources, while the method in Reference [40] is more affected by the number of array elements. However, the proposed method should have better performance than the EACDM method and the method in Reference [40] at low SNR. The EACDM method uses approximate calculations in the derivation process, but this approximation will have a large deviation when the SNR is lower than 10dB. The method in Reference [40] performs eigen-decomposition of the array covariance matrix during the derivation process to separate the signal subspace and the noise subspace. Under the low SNR, the separation effect will be worse.

In addition, the gain errors estimation accuracy of the proposed method is not affected by the magnitude of the phase error and the number of elements. According to Equation (4), it can be easily shown that

(21) $$abs\left({\left[a^{*}\left(\hat{\theta }\right)\right]}_{i}\right)=1$$

Therefore, the gain errors estimates in Equation (20) can be rewritten as

(22) $$\rho_{i}=abs\left({\left[\hat{b}\left(\theta \right)\right]}_{i}\cdot {\left[a^{*}\left(\hat{\theta }\right)\right]}_{i}\right)=abs\left({\left[\hat{b}\left(\theta \right)\right]}_{i}\right).$$

From Equation (22), the accuracy of the gain errors estimation is independent of the estimation accuracy of the DOA, which increases as the number of sensor elements increases. Thus, the accuracy of the gain errors estimation is independent of the number of sensor elements and only relates to the estimation accuracy of $\hat{b}\left(\theta \right)$.

## 5. Numerical Simulations

**Figure 2:**
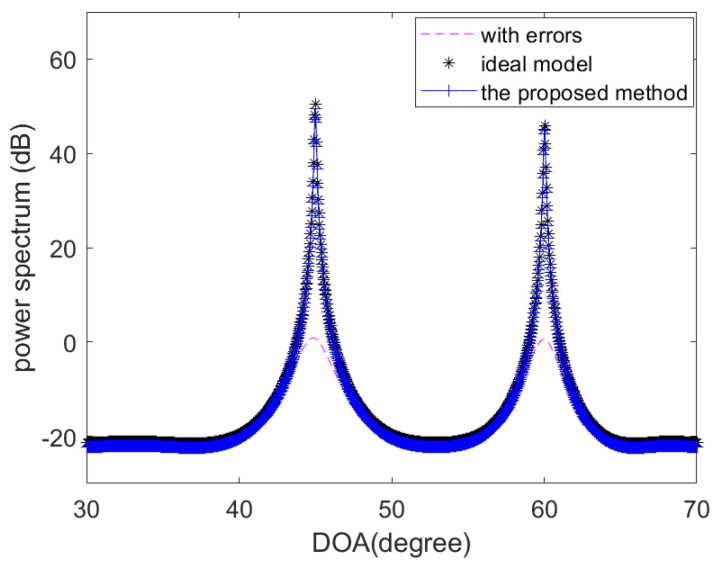
The MUSIC spatial spectrum using the ideal model, the model calibrated by the estimated errors (using the proposed method) and the uncalibrated model.

Being the same as Reference [16], in the following experiments, the gain errors and phase errors are randomly generated, and the maximum gain error is less than 5% (from unity), and the maximum phase error is less than 1.57rad. The distance between the *i*-th element and the first element is given by $P={\left[d_{2},d_{3},\ldots d_{M}\right]}^{T}={\left[0.55\lambda ,0.45\lambda ,\ldots ,0.45\lambda ,0.55\lambda \right]}^{T}$. The central wavelength of incident signals is λ. In the first experiment, consider a non-uniform linear array consisting of 24 omni-directional sensor elements. The two equal-power narrowband signal sources are located at $\theta_{1}=45^{\circ }$ and $\theta_{2}=60^{\circ }$. The SNR is 10dB. The number of time samples is 800 and the searching step is $0.1^{\circ }$. Then, we obtain the MUSIC spatial spectrum using the ideal model, the model calibrated by the estimated errors (using the proposed method) and the uncalibrated model. The actual and estimated values of the gain-phase errors of the first eight elements are compared in Table 1. It can be seen from Table 1 and Table 2 and Figure 2 that the proposed method has the good performance to estimate the sensor gain-phase errors accurately.

**Figure 3:**
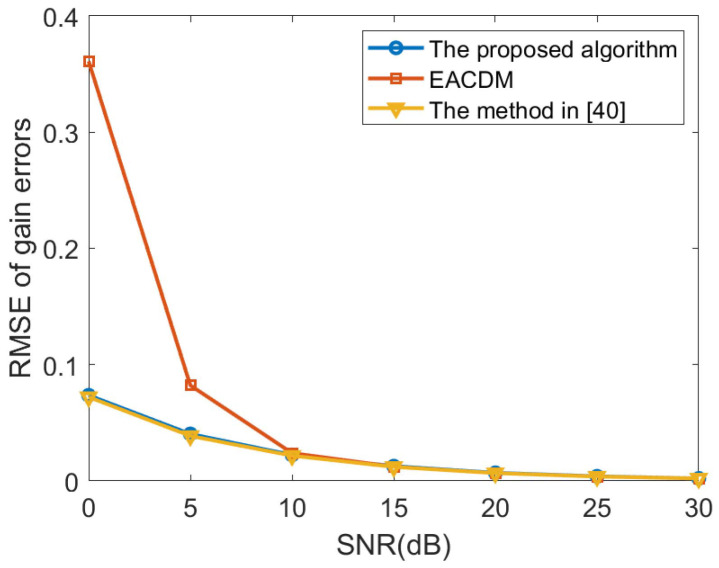
Root mean square error (RMSE) curves of gain errors estimates versus the SNR.

**Figure 4:**
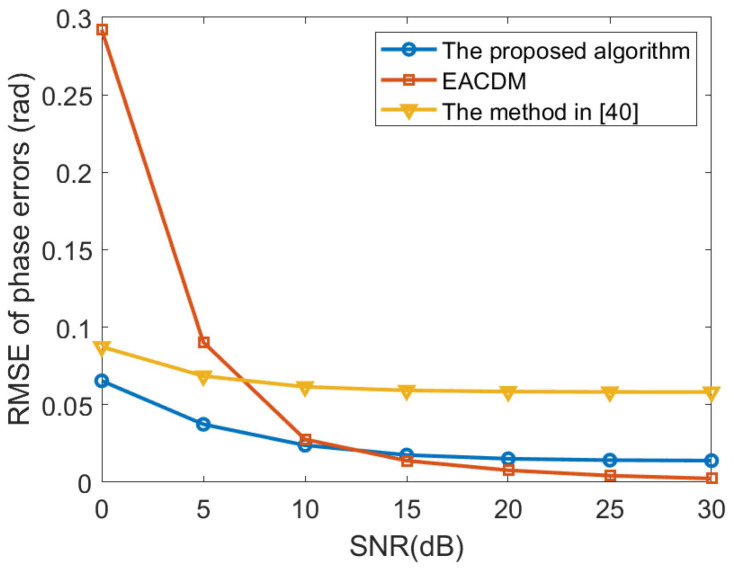
RMSE curves of phase errors estimates versus the SNR.

In the second experiment, the proposed method, the method in Reference [40] and the EACDM method in Reference [16] are compared when the SNR level changes. Consider a non-uniform linear array consisting of 12 omni-directional sensor elements. The distance between the *i*-th element and the first element is given by $P={\left[d_{2},d_{3},\ldots d_{M}\right]}^{T}={\left[0.55\lambda ,0.45\lambda ,\ldots ,0.45\lambda ,0.55\lambda \right]}^{T}$. The source is located at the direction $\theta_{1}=20^{\circ }$. The number of time samples is 200 and the searching step is $0.01^{\circ }$. We performed 500 independent Monte Carlo experiments, the root mean square error (RMSE) curves of gain and phase errors estimates are illustrated in Figure 3 and Figure 4 Compared with the EACDM method, the proposed method has better performance at low SNR.

**Figure 5:**
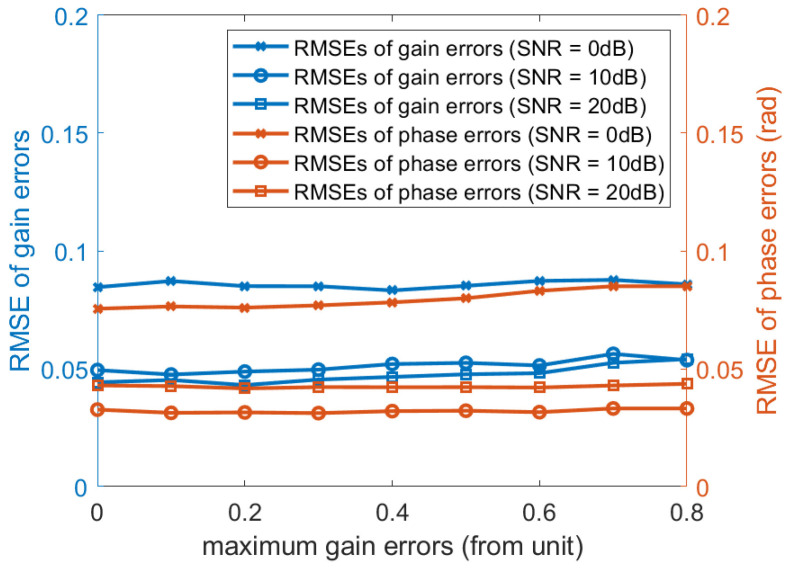
RMSE curves of gain and phase errors estimates versus the maximum gain errors (from unit).

In the third experiment, we examine the influence of the maximum gain errors (from unit) to verify that the proposed algorithm performs independently of gain errors. Consider a non-uniform linear array consisting of 20 omni-directional sensor elements. The distance between the *i*-th element and the first element is given by $P={\left[d_{2},d_{3},\ldots d_{M}\right]}^{T}={\left[0.55\lambda ,0.45\lambda ,\ldots ,0.45\lambda ,0.55\lambda \right]}^{T}$. The two sources are located at the direction $\theta_{1}=20^{\circ }$ and $\theta_{2}=50^{\circ }$, respectively. The number of time samples is 200 and the searching step is $0.1^{\circ }$. We performed 500 independent Monte Carlo experiments, Figure 5 illustrate the RMSE curves of gain and phase errors estimates. It can be seen that the RMSE curves are changeless when the maximum gain errors changes.

**Figure 6:**
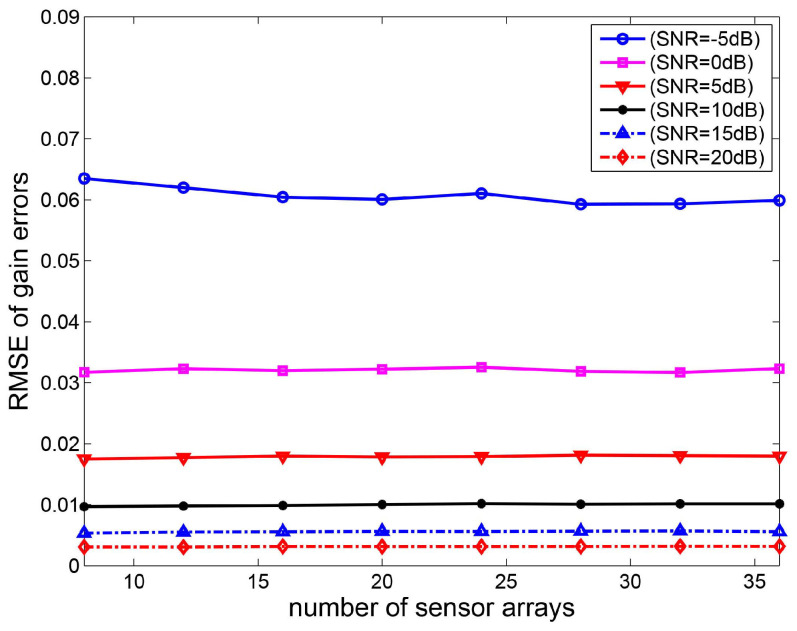
RMSE curves of gain errors estimates versus the number of sensor arrays.

**Figure 7:**
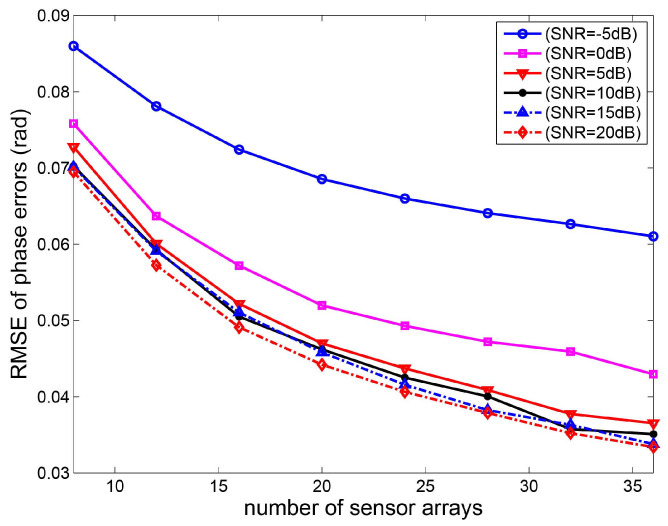
RMSE curves of phase errors estimates versus the number of sensor arrays.

In the fourth experiment, the root mean square error curves of gain-phase errors estimates versus the number of sensor arrays are shown. Consider a non-uniform linear array and the distance between the *i*-th element and the first element is given by $P={\left[d_{2},d_{3},\ldots d_{M}\right]}^{T}={\left[0.55\lambda ,0.45\lambda ,\ldots ,0.45\lambda ,0.55\lambda \right]}^{T}$. The two sources are located at the direction $\theta_{1}=20^{\circ }$ and $\theta_{2}=50^{\circ }$, respectively. Based on 2000 Monte Carlo experiments, the RMSE curves of gain-phase errors estimates versus the number of sensor elements are depicted in Figure 6 and Figure 7. It can be seen that the phase errors estimation accuracy increases as the number of sensor arrays increases. This is because the covariance of the DOA estimate converges to zero as M→∞. However, the RMSE of gain errors estimates is changeless as the number of sensor arrays increases.

## 6. Conclusions

This paper presents a blind calibration algorithm for linear arrays, which can estimate the gain-phase errors and DOA simultaneously. A spatial spectrum is constructed based on blind source separation. Through a one-dimensional search of spatial spectrum, DOA is obtained first and then gain-phase errors are estimated. The proposed algorithm does not need a joint iteration and avoids the problem of local optimization. At the same time, the algorithm performs independently of the gain error. No matter how large the gain error is, the proposed algorithm can always maintain good performance.

## Figures and Tables

**Table 1:** Actual and Estimated Values of Gain Errors.

Element Number	1	2	3	4
Actual Values	1.0000	1.0186	1.0299	0.9961
Estimated Values	1.0000	1.0175	1.0336	0.9936
Estimated Bias	0	0.0009	0.0037	0.0025
Element number	5	6	7	8
Actual Values	1.0099	1.0356	1.0139	1.0146
Estimated Values	1.0145	1.0316	1.0116	1.0154
Estimated Bbias	0.0046	0.0040	0.0023	0.0008

**Table 2:** Actual and Estimated Values of Phase Errors.

Element Number	1	2	3	4
Actual Values	0	0.9595	1.3287	−1.3940
Estimated Values	0	0.9633	1.3296	−1.3866
Estimated Bias	0	0.0038	0.0009	0.0074
Element Number	5	6	7	8
Actual Values	0.8843	1.1036	−0.9440	−1.0310
Estimated Values	0.8753	1.0985	−0.9537	−1.0214
Estimated Bias	0.0090	0.0051	0.0097	0.0096
